# Incidence and risk factors of admission deep venous thrombosis in nonagenarians and centenarians with intertrochanteric fracture: a retrospective study

**DOI:** 10.1186/s13018-023-04032-9

**Published:** 2023-08-02

**Authors:** Tao Wang, Shuo Yang, Zhiqian Wang, Junfei Guo, Zhiyong Hou

**Affiliations:** 1 Department of Orthopaedic Surgery, The Third Hospital of Hebei Medical University, Shijiazhuang, Hebei People’s Republic of China; 2 Orthopaedic Research Institute of Hebei Province, Shijiazhuang, 050051 Hebei People’s Republic of China; 3 Department of Joint Surgery, Honghui Hospital, Xi’an Jiaotong University, Xi’an, People’s Republic of China; 4 NHC Key Laboratory of Intelligent Orthopaedic Equipment (The Third Hospital of Hebei Medical University), Shijiazhuang, People’s Republic of China

**Keywords:** Intertrochanteric fracture, Nonagenarians and centenarians, Deep venous thrombosis, D-dimer

## Abstract

**Purpose:**

The objective of this study was to identify the risk factors for admission deep venous thrombosis (aDVT) and proximal aDVT in nonagenarians and centenarians with intertrochanteric fracture (IF).

**Methods:**

We collected statistics on nonagenarians and centenarians with IF admitting from January 2010 to October 2022. Patients with aDVT were considered as the aDVT group and those without aDVT as the non-aDVT group. Additionally, we also conducted a subgroup analysis based on the location of aDVT to investigate the predictors of proximal aDVT. The demographics, comorbidities and admission laboratory examinations of patients were computed by univariate analysis, logistic regression analysis, and receiver operating characteristic (ROC) curve analysis.

**Results:**

In our study, the rate of aDVT (109 of 318) was 34.3%, and 5.7% (18 of 318) of patients had proximal aDVT. Logistic regression analysis showed that female patients and a high level of D-dimer were risk factors for aDVT. Similarly, hypoproteinemia and a high level of D-dimer were found to be risk factors for proximal aDVT. ROC curve analysis indicated the cut-off values of D-dimer to predict the aDVT and proximal aDVT were 1.28 mg/L and 1.485 mg/L, respectively.

**Conclusions:**

Our findings investigated the risk factors of aDVT and proximal aDVT in nonagenarians and centenarians with IF and identified the cut-off values of D-dimer, helping us assess the risk of aDVT and proximal aDVT to manage early targeted interventions.

*Level of evidence* III.

## Introduction

Intertrochanteric fracture (IF) is a common osteoporosis-related fracture in the elders that affects 10–20% of overall fractures [1] and occupies 50–65% of hip fractures [2]. Prior study has reported that the number of hip fracture patients will up to 6.26 million by 2050, and IF accounts for more than half of hip fractures [3–5]. With the development of medical care, the number of patients over 90 years old (nonagenarians and centenarians) has rapidly increased, bringing enormous challenges for clinicians and great burdens for families and societies because the aging population with IF can cause mortality and morbidity [6]. Our previous findings showed 7.6%, 13.9%, and 28.5% mortality in nonagenarians and centenarians with IF at 6-month, 1-year, and 2-year follow-up, respectively [7].

Admission deep venous thrombosis (aDVT) is one of the most complications after IF that affects 8.0–34.9% of the older patients [8–10] due to a hypercoagulable state and immobilization. Zhao [11] has demonstrated that delayed surgery, hypoproteinemia, three or more comorbidities, and a D-dimer level > 1.59 mg/L were related to preoperative deep venous thrombosis (DVT). However, Kobayashi [12] reported that female patients, advanced age, delayed time from injury to admission and from injury to surgery, and kidney disease were risk factors for preoperative DVT. Our recent meta-analysis indicated that many factors were associated with preoperative DVT [8] in patients with hip fractures. Proximal DVT, defined as popliteal and/or more proximal DVT, may lead to fatal outcomes, such as pulmonary embolism (PE) [13, 14]. To our knowledge, limited studies focus on the risk factors of aDVT and proximal aDVT in nonagenarians and centenarians with IF. This is the first study concerning nonagenarians and centenarians to investigate the risk factors for aDVT and proximal aDVT after IF. Our primary purpose is to identify the predictors related to aDVT in nonagenarians and centenarians with IF, and our second goal is to find the risk factors for proximal aDVT.

## Patients and methods

### Ethics statement

This retrospective study was approved by the Institutional Review Board of our hospital before collecting data.

### Patients

We included 318 nonagenarians and centenarians with IF between January 2010 and October 2022 in our hospital. All patients received color Doppler ultrasound to detect DVT at admission. According to the location of aDVT, we defined thrombosis in popliteal vein or more proximal as the proximal aDVT and regraded thrombosis in the muscle veins, tibial veins or peroneal veins as the distal aDVT. Based on the detection of aDVT or not, we divided these patients into the aDVT group and the non-aDVT group. Similarly, according to the location of aDVT, we divided aDVT patients into the distal group (DG) and the proximal group (PG). The inclusion criteria were as follows: (1) nonagenarians and centenarians; (2) patients with fresh IF (< 21 days); and (3) no comorbidity was present at the time of admission; the exclusion criteria: (1) patients with a history of IF; (2) patients with a history of DVT; (3) patients with pathological fractures; (4) patients with open fractures; and (5) incomplete data.

The demographics, comorbidities, and admission laboratory examinations of patients were collected in this study. The demographics data included age, gender, time from injury to hospital, body mass index (< 24, 24–28, and > 28 kg/m^2^), type of fracture, injury side. Comorbidities consist of anemia, electrolyte disturbance, dementia, pneumonia, arteriosclerosis, hypoproteinemia, arrhythmia, heart valve disease, heart failure, heart infarction, diabetes, intracerebral hemorrhage, coronary heart disease, hypertension, and cerebral infarction. Admission laboratory examinations covered prothrombin time (PT), international normalized ratio (INR), fibrinogen (FIB), activated partial thromboplastin time (APTT), thrombin time (TT), D-dimer, antithrombin III (AT III), white blood cell (WBC), neutrophil (NEU), lymphocyte (LYM), monocyte (MON), red blood cell (RBC), hemoglobin (HGB), platelet (PLT), total protein (TP), albumin (ALB), globulin (GLOB), ALB/GLOB, creatine kinase (CK), creatine Kinase Isoenzyme (CKMB), and c-reactive protein (CRP).

### Statistics

We utilized SPSS (version 21.0 SPSS Inc., Chicago, IL) and regarded *p* < 0.05 as statistical significance. Regarding continuous variables, if data met normality criteria, all measurement data were presented as the mean ± SD (standard deviation) using t-test, but if not, the Mann–Whitney U test was used to perform statistical analysis between groups. For count data, the chi-square test was used for data analysis. Furthermore, to identify the best predictors of aDVT, we used binary logistic regression analysis to detect independent predictors of aDVT and proximal aDVT. Additionally, receiver operator characteristic (ROC) curve analysis was used to identify the cutoff values for continuous variables, such as D-dimer. The area under the ROC curve (AUC) was used to determine the diagnostic ability, ranging from 0 to 100%, with more area meaning better ability. We choose the cut-of values for continuous variables by the maximum Youden index (sensitivity + specificity − 1) in the ROC curve analysis.

## Results

A total of 318 nonagenarians and centenarians with IF were included in this study: 209 patients without aDVT and 109 patients with aDVT. The rate of aDVT was 34.3%. Among aDVT patients, eight-four cases were found in calf muscular venous thrombosis, seven cases in tibial vein thrombosis, six cases in popliteal vein, and twelve cases in more proximal vein. Thus, the rate of proximal aDVT and distal aDVT were 5.7% (18 of 109 patients) and 28.6% (91 of 109 patients), respectively.

**Fig. 1:**
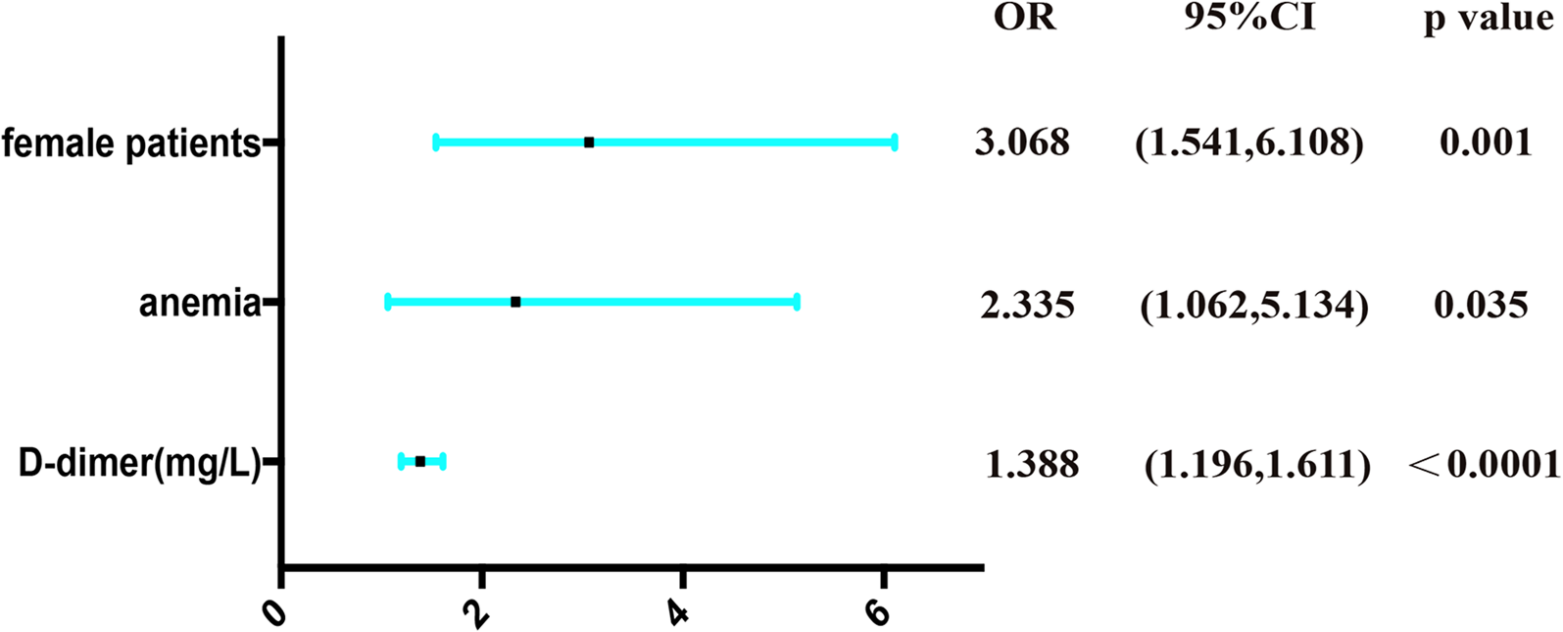
OR, 95% CI, and *p* value for independent risk factors in the multivariable logistic regression analysis of aDVT. OR — odd ratio; CI — confidence interval; DVT — admission deep venous thrombosis

**Fig. 2:**
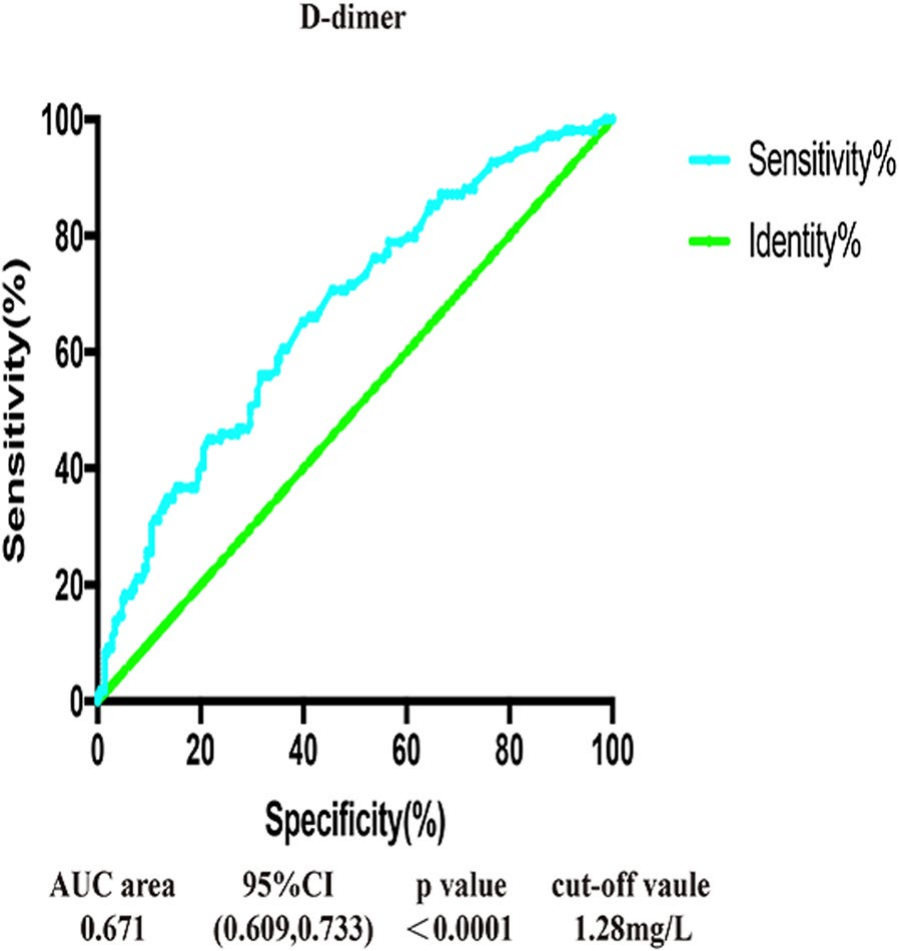
AUC area, 95% CI, *p* value and cut-off value for independent risk factors in the ROC curve analysis of aDVT. OR — odd ratio; CI — confidence interval; DVT = admission deep venous thrombosis; ROC — receiver operating characteristic

As presented in Table 1, female patients (*p* = 0.01) and patients with a history of anemia (*p* = 0.024) and arteriosclerosis (*p* = 0.03) were found to be associated with the risk of aDVT. The level of D-dimer (*p* < 0.0001) was significantly higher, but the level of TP (*p* = 0.046) was markedly lower in the aDVT group than in the non-aDVT group. We also found the normal range of A/G (*p* = 0.031) related to aDVT. Logistic regression analysis indicated that female patients [*p* = 0.001, OR 3.068, 95%CI (1.541, 6.108)], patients with a history of anemia [*p* = 0.035, OR 2.335, 95%CI (1.062, 5.134)], and a higher level of D-dimer [*p* < 0.0001, OR 1.388, 95%CI (1.196, 1.611)] were independent risk factors of aDVT in nonagenarians and centenarians with IF (Fig. 1). ROC curve analysis showed that the level of D-dimer [*p* < 0.0001, AUC area = 0.671, 95%CI (0.609, 0.733)] was an independent predictor of aDVT in nonagenarians and centenarians with IF and identified that the cut-off value of D-dimer was 1.28 mg/L (sensitivity = 0.651; specificity = 0.603) (Fig. 2).

**Table 1:** Possible factors may be associated with admission deep venous thrombosis in two groups

Characteristics	DVT group (*n* = 109)	Non-DVT group (*n* = 209)	*p*
Age, years	92.1 ± 2.6	92.6 ± 2.8	0.08
Gender (*n*, %)			*0.01*
Male	16 (14.7%)	65 (31.1%)	
Female	93 (85.3%)	144 (68.9%)	
Body mass index (kg/m^2^)	22.22 (19.69–24.03)	21.48 (19.53–25.1)	0.275
≤ 24	79 (72.5%)	146 (69.9%)	0.838
24–28	22 (20.2%)	44 (21.1%)	
> 28	8 (7.3%)	19 (9.0%)	
Time from injury to hospital (hours, *n*, %)			0.326
≤ 12	16 (14.7%)	38 (7.3%)	
13–24	9 (8.3%)	26 (7.3%)	
> 24	84 (77.0%)	145 (7.3%)	
Fracture type (*n*, %)			0.147
AO A1.1–2.1	62 (56.9%)	101 (48.3%)	
AO A2.2–3.1	47 (43.1%)	108 (51.7%)	
Injury side (*n*, %)			0.377
Left	47 (43.1%)	101 (48.3%)	
Right	62 (56.9%)	108 (51.7%)	
*Comorbidities*			
Anemia (*n*, %)			*0.024*
Yes	95 (87.2%)	160 (76.6%)	
No	14 (12.8%)	49 (23.4%)	
Electrolyte disturbance (*n*, %)			0.636
Yes	21 (19.3%)	45 (21.5%)	
No	88 (80.7%)	164 (78.5%)	
Dementia (*n*, %)			0.85
Yes	9 (8.3%)	16 (7.7%)	
No	100 (91.7%)	193 (92.3%)	
Pneumonia (*n*, %)			0.71
Yes	19 (17.4%)	40 (19.1%)	
No	90 (82.6%)	169 (80.9%)	
Arteriosclerosis (*n*, %)			*0.03*
Yes	19 (17.4%)	19 (9.1%)	
No	90 (82.6%)	190 (90.9%)	
Hypoproteinemia (*n*, %)			0.454
Yes	33 (30.3%)	55 (26.3%)	
No	76 (69.7%)	154 (73.7%)	
Arrhythmia (*n*, %)			0.842
Yes	24 (22.0%)	44 (21.1%)	
No	85 (78.0%)	165 (78.9%)	
Heart valve disease (*n*, %)			0.111
Yes	8 (7.3%)	7 (3.3%)	
No	101 (92.7%)	202 (96.7%)	
Heart failure (*n*, %)			*0.018*
Yes	6 (5.5%)	30 (14.4%)	
No	103 (94.5%)	179 (85.6%)	
Heart infarction (*n*, %)			0.961
Yes	4 (3.7%)	6 (2.9%)	
No	105 (96.3%)	203 (97.1%)	
Diabetes (*n*, %)			0.895
Yes	12 (11.0%)	22 (10.5%)	
No	97 (89.0%)	187 (89.5%)	
Intracerebral hemorrhage (*n*, %)			0.356
Yes	0 (0%)	4 (1.9%)	
No	109 (100%)	205 (98.1%)	
Coronary heart disease (*n*, %)			0.578
Yes	27 (24.8%)	46 (22.0%)	
No	82 (75.2%)	163 (78.0%)	
Hypertension (*n*, %)			0.69
Yes	40 (36.7%)	72 (34.4%)	
No	69 (63.3%)	137 (65.6%)	
Cerebral infarction (*n*, %)			0.692
Yes	29 (26.6%)	60 (28.7%)	
No	80 (73.4%)	149 (71.3%)	
*Laboratory examinations*			
PT (s)	12 (11.3–12.7)	12 (11.23–12.9)	0.716
Normal (9.0–12.5 s)	79 (72.5%)	139 (66.5%)	0.276
Abnormal	30 (27.5%)	70 (33.5%)	
INR (s)	1.08 (1.02–1.12)	1.07 (1.01–1.14)	0.812
Normal (0.8–1.4 s)	109 (100%)	207 (99.0%)	0.782
Abnormal	0 (0%)	2 (1.0%)	
FIB (g/L)	3.47 (3.04–4.05)	3.54 (3.00–4.08)	0.95
Normal (2–4 g/L)	77 (70.6%)	146 (69.9%)	0.884
Abnormal	32 (29.4%)	63 (30.1%)	
APTT (s)	28.9 (27.0–31.45)	29.9 (27.35–33.65)	0.21
Normal (28–42 s)	66 (60.6%)	133 (63.6%)	0.589
Abnormal	43 (39.4%)	76 (36.3%)	
TT (s)	15.2 (14.15–16.15)	15.2 (14.2–16.35)	0.369
Normal (12–17 s)	97 (89.0%)	169 (80.9%)	0.063
Abnormal	12 (11.0%)	40 (19.1%)	
D-dimer (mg/L)	1.85 (1.07–3.65)	1.1 (0.7–2.2)	**< 0.0001**
≤ 1.28 mg/L	38 (34.9%)	126 (60.3%)	**< 0.0001**
> 1.28 mg/L	71 (65.1%)	83 (39.7%)	
AT III (%)	84 (73.5–96.0)	86 (74.55–93.0)	0.626
Normal (80–120%)	65 (59.6%)	131 (62.7%)	0.596
Abnormal	44 (40.4%)	78 (37.3%)	
WBC (10^9^/L)	7.81 (6.48–9.50)	8.02 (6.67–9.80)	0.768
Normal (3.5–9.5 10^9^/L)	79 (72.5%)	145 (69.4%)	0.565
Abnormal	30 (27.5%)	64 (30.6%)	
NEU (10^9^/L)	5.97 (4.80–7.64)	6.1 (4.82–7.64)	0.995
Normal (1.8–6.3 10^9^/L)	63 (57.8%)	113 (54.1%)	0.525
Abnormal	46 (42.2%)	96 (45.9%)	
LYM (10^9^/L)	1.08 (0.83–1.39)	1.13 (0.87–1.50)	0.18
Normal (1.1–3.2 10^9^/L)	53 (48.6%)	109 (52.2%)	0.55
Abnormal	56 (51.4%)	100 (47.8%)	
MON (10^9^/L)	0.64 (0.44–0.99)	0.63 (0.46–0.79)	0.732
Normal (0.1–0.6 10^9^/L)	50 (45.9%)	94 (45.0%)	0.879
Abnormal	59 (54.1%)	115 (55.0%)	
RBC (10^12^/L)	3.12 (2.75–3.55)	3.2 (2.80–3.64)	0.249
Normal (3.8–5.1 10^12^/L)	16 (14.7%)	34 (16.3%)	0.712
Abnormal	93 (85.3%)	175 (83.7%)	
HGB (g/L)	97.7 ± 16.3	99.2 ± 17.3	0.45
Normal (115–150 g/L)	14 (12.8%)	41 (19.6%)	0.130
Abnormal	95 (87.2%)	168 (80.4%)	
PLT (10^9^/L)	164.5 (132.0–205.2)	170.3 (131.7–208.0)	0.747
Normal (125–350 10^9^/L)	84 (77.1%)	155 (74.2%)	0.57
Abnormal	25 (22.9%)	54 (25.8%)	
TP (g/L)	57.8 (53.86–61.65)	59.02 (55.06–62.98)	**0.046**
Normal (60–80 g/L)	38 (34.9%)	86 (41.1%)	0.275
Abnormal	71 (65.1%)	123 (58.9%)	
ALB (g/L)	34.23 (32.0–36.35)	34.64 (32.35–37.7)	0.175
Normal (35–55 g/L)	45 (41.3%)	100 (47.8%)	0.265
Abnormal	64 (58.7%)	109 (52.2%)	
GLOB (g/L)	23.8 ± 4.6	24.6 ± 5.3	0.154
Normal (20–30 g/L)	82 (75.2%)	136 (65.1%)	0.064
Abnormal	27 (24.8%)	73 (34.9%)	
A/G	1.47 (1.29–1.72)	1.42 (1.19–1.66)	0.199
Normal (1.0–2.5)	103 (95.0%)	181 (86.6%)	*0.031*
Abnormal	6 (5.0%)	28 (13.4%)	
CK (U/L)	113 (62.4–227.1)	92.65 (54.75–185.33)	0.208
Normal (25–130U/L)	56 (51.4%)	124 (59.3%)	0.174
Abnormal	53 (48.6%)	85 (40.7%)	
CKMB (U/L)	10.65 (7.7–14.87)	11.0 (8.00–15.83)	0.689
Normal (3–20U/L)	96 (88.1%)	178 (85.2%)	0.476
Abnormal	13 (11.9%)	31 (14.8%)	
CRP (mg/L)	41.59 (15.36–73.01)	37.7 (16.79–72.11)	0.822
Normal (< 10 mg/L)	13 (11.9%)	31 (14.8%)	0.476
Abnormal	96 (88.1%)	178 (85.2%)	

Bold and italics just remind us the significant variables

Values are presented as the number (%) or the median (interquartile range). BMI are presented as mean or standard deviation

*DVT* deep venous thrombosis, *PT* prothrombin time, *INR* international normalized ratio, *FIB* fibrinogen, *APTT* activated partial thromboplastin time, *TT* thrombin time, *AT III* antithrombin III, *WBC* white blood cell, *NEU* neutrophil, *LYM* lymphocyte, *MON* monocyte, *RBC* red blood cell, *HGB* hemoglobin, *PLT* platelet, *TP* total protein, *ALB* albumin, *GLOB* globulin, *CK* creatine kinase, *CKMB* creatine Kinase Isoenzyme, *CRP* c-reactive protein

*p < 0.05, statistical significance

**Fig. 3:**
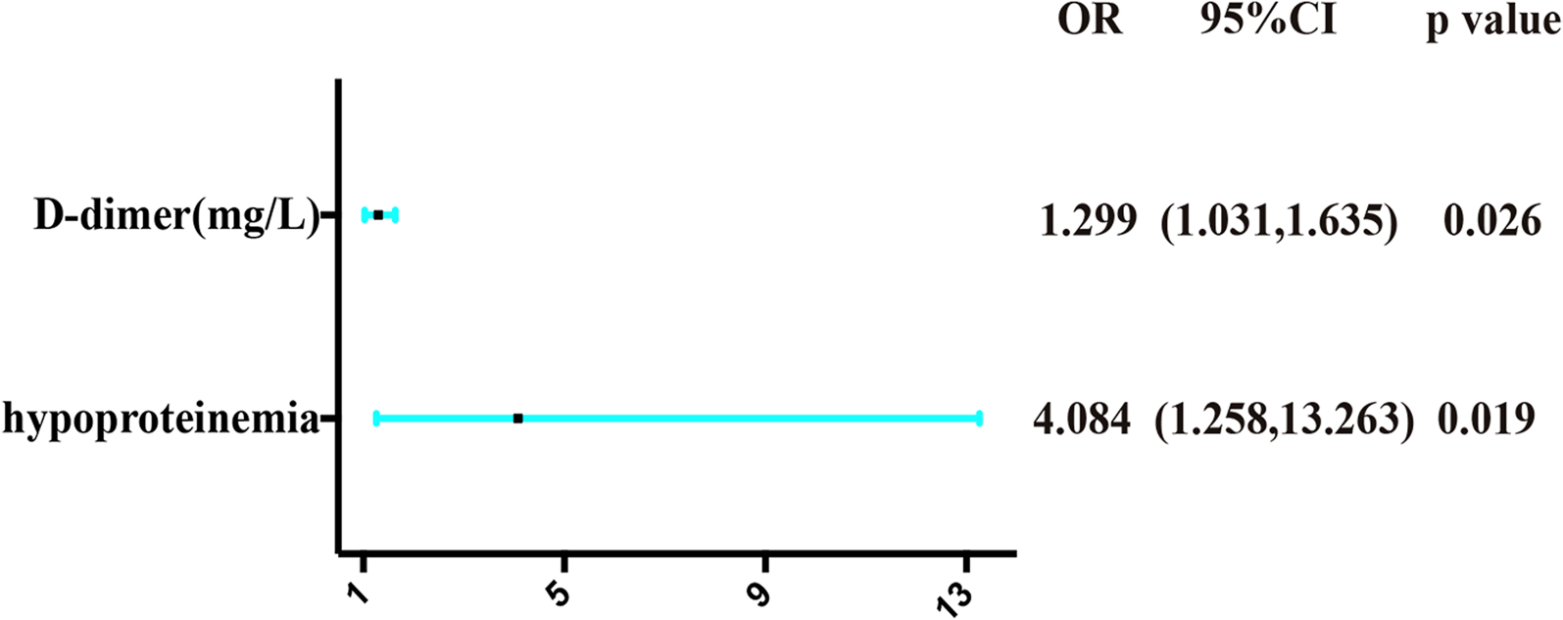
OR, 95% CI, and *p* value for independent risk factors in the multivariable logistic regression analysis of proximal aDVT. OR — odd ratio; CI —confidence interval; DVT —admission deep venous thrombosis

**Fig. 4:**
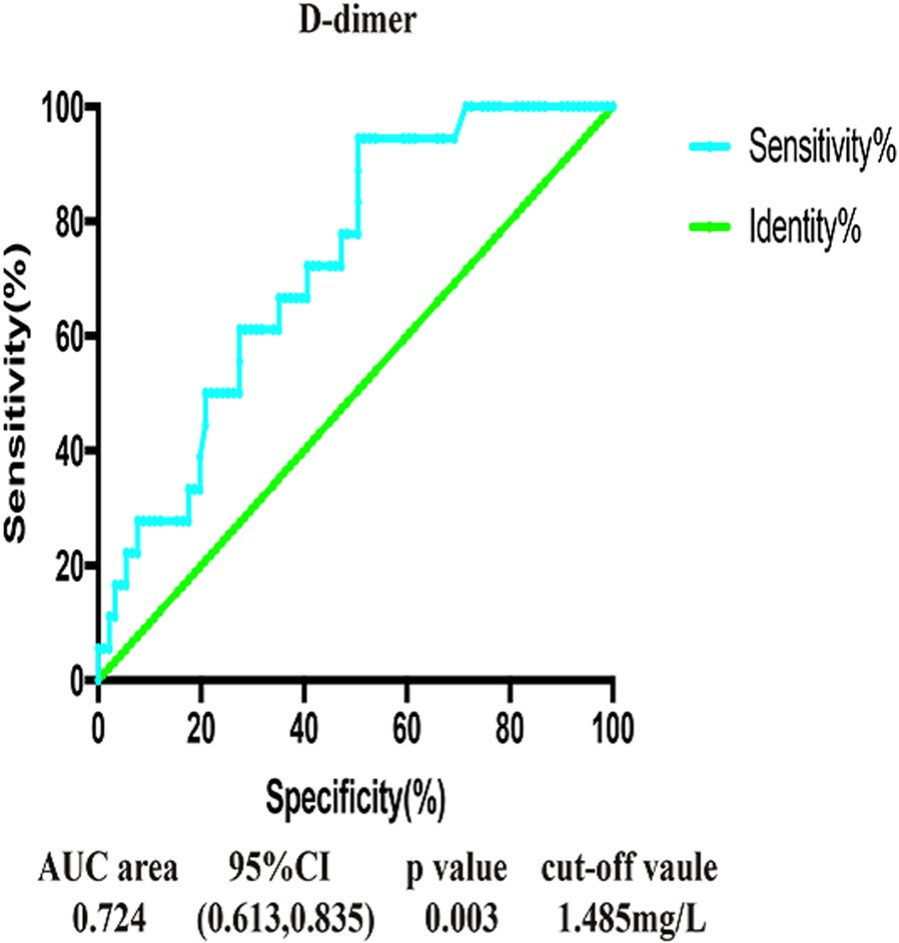
AUC area, 95% CI, *p* value and cut-off value for independent risk factors in the ROC curve analysis of proximal aDVT. OR — odd ratio; CI — confidence interval; DVT — admission deep venous thrombosis; ROC — receiver operating characteristic

As shown in Table 2, patients with a history of hypoproteinemia (*p* = 0.011) was found to be associated with a higher risk of proximal aDVT. The level of D-dimer (*p* = 0.003) was significantly higher in the PG than in the DG. We also found the normal range of CKMB (*p* < 0.0001) related to proximal aDVT. Logistic regression analysis indicated that patients with a history of hypoproteinemia [*p* = 0.019, OR 4.084, 95%CI (1.258, 13.263)] and a higher level of D-dimer [*p* = 0.026, OR 1.299, 95%CI (1.031, 1.635)] were independent risk factors for proximal aDVT in nonagenarians and centenarians with IF (Fig. 3). ROC curve analysis showed that the level of D-dimer [*p* = 0.003, AUC area = 0.724, 95%CI (0.613, 0.835)] was an independent predictor of aDVT in nonagenarians and centenarians with IF and identified the cut-off value of D-dimer as 1.485 mg/L (sensitivity = 0.944; specificity = 0.495) (Fig. 4).

**Table 2:** Possible factors may be associated with admission deep venous thrombosis in two groups

Characteristics	Proximal DVT (*n* = 18)	Distal DVT (*n* = 91)	*p*
Age, years	90.5 (90.0–92.25)	91 (91–93)	0.067
Gender (*n*, %)			1.000
Male	3 (16.7%)	13 (14.3%)	
Female	15 (83.3%)	78 (85.7%)	
Body mass index (kg/m^2^)	22.6 (19.8–27.3)	23.0 (19.4–23.9)	0.465
≤ 24	10 (55.6%)	69 (75.8%)	1.000
24–28	6 (33.3%)	16 (17.6%)	
> 28	2 (11.1%)	6 (6.6%)	
Time from injury to hospital (hours, *n*, %)			0.566
≤ 12	4 (22.2%)	12 (13.2%)	
13–24	1 (11.1%)	9 (9.9%)	
> 24	13 (72.2%)	70 (76.9%)	
Fracture type (*n*, %)			1.000
AO A1.1–2.1	9 (50.0%)	43 (47.3%)	
AO A2.2–3.1	9 (50.0%)	48 (52.7%)	
Injury side (*n*, %)			1.000
Left	8 (44.4%)	39 (42.9%)	
Right	10 (55.6%)	52 (57.1%)	
*Comorbidities*			
Anemia (*n*, %)			0.885
Yes	15 (83.3%)	80 (87.9%)	
No	3 (16.7%)	11 (12.1%)	
Electrolyte disturbance (*n*, %)			0.983
Yes	4 (22.2%)	17 (18.7%)	
No	14 (77.8%)	74 (81.3%)	
Dementia (*n*, %)			1.000
Yes	1 (5.6%)	8 (8.8%)	
No	17 (94.4%)	83 (91.2%)	
Pneumonia (*n*, %)			0.265
Yes	1 (5.6%)	18 (19.8%)	
No	17 (94.4%)	73 (80.2%)	
Arteriosclerosis (*n*, %)			1.000
Yes	3 (16.7%)	16 (17.6%)	
No	15 (83.3%)	75 (82.4%)	
Hypoproteinemia (*n*, %)			**0.011**
Yes	10 (55.6%)	23 (25.3%)	
No	8 (44.4%)	68 (74.7%)	
Arrhythmia (*n*, %)			0.362
Yes	2 (11.1%)	22 (24.2%)	
No	16 (88.9%)	69 (75.8%)	
Heart valve disease (*n*, %)			0.417
Yes	0 (0%)	8 (8.8%)	
No	18 (100%)	83 (91.2%)	
Heart failure (*n*, %)			0.565
Yes	2 (11.1%)	4 (4.4%)	
No	16 (88.9%)	87 (95.6%)	
Heart infarction (*n*, %)			1.000
Yes	1 (5.6%)	3 (3.3%)	
No	17 (94.4%)	88 (96.7%)	
Diabetes (*n*, %)			1.000
Yes	2 (11.1%)	10 (11.0%)	
No	16 (88.9%)	81 (89.0%)	
Intracerebral hemorrhage (*n*, %)			NA
Yes	0 (0%)	0 (0%)	
No	18 (100%)	91 (100%)	
Coronary heart disease (*n*, %)			1.000
Yes	4 (22.2%)	23 (25.3%)	
No	14 (77.8%)	68 (74.7%)	
Hypertension (*n*, %)			0.455
Yes	8 (44.4%)	32 (35.2%)	
No	10 (55.6%)	59 (64.8%)	
Cerebral infarction (*n*, %)			0.678
Yes	6 (33.3%)	23 (25.3%)	
No	12 (66.7%)	68 (74.7%)	
*Laboratory examinations (n, %)*			
PT (s)	12.3 (11.7–13.2)	11.9 (11.3–12.5)	0.104
Normal (9.0–12.5 s)	10 (55.6%)	69 (75.8%)	0.141
Abnormal	8 (44.4%)	22 (24.2%)	
INR (s)	1.08 (1.05–11.6)	1.07 (1.01–1.11)	0.164
Normal (0.8–1.4 s)	18 (100%)	91 (100%)	NA
Abnormal	0 (0%)	0 (0%)	
FIB (g/L)	3.29 (2.93–3.63)	3.58 (3.04–4.06)	0.344
Normal (2–4 g/L)	15 (83.3%)	62 (68.1%)	0.196
Abnormal	3 (16.7%)	29 (31.9%)	
APTT (s)	29.3 (27.3–33.6)	28.9 (26.8–31.1)	0.278
Normal (28–42 s)	12 (66.7%)	54 (59.3%)	0.561
Abnormal	6 (33.3%)	37 (40.7%)	
TT (s)	15.0 (14.3–15.9)	15.3 (14.0–16.2)	0.719
Normal (12–17 s)	17 (94.4%)	81 (89.0%)	0.786
Abnormal	1 (5.6%)	10 (11.0%)	
D-dimer (mg/L)	3.37 (1.77–5.86)	1.70 (0.95–3.36)	**0.003**
≤ 1.485 mg/L	1 (5.6%)	44 (48.4%)	**0.001**
> 1.485 mg/L	17 (94.4%)	47 (51.6%)	
AT III (%)	81.5 (69.8–92.0)	85.0 (75.0–96.0)	
Normal (80–120%)	10 (55.6%)	55 (60.4%)	0.700
Abnormal	8 (44.4%)	36 (39.6%)	
WBC (10^9^/L)	7.83 (5.49–10.27)	7.73 (6.62–9.24)	0.621
Normal (3.5–9.5 10^9^/L)	11 (61.1%)	68 (74.7%)	0.372
Abnormal	7 (38.9%)	23 (25.3%)	
NEU (10^9^/L)	5.69 (3.82–8.37)	5.98 (4.97–7.53)	0.470
Normal (1.8–6.3 10^9^/L)	10 (55.6%)	53 (58.2%)	**0.833**
Abnormal	8 (44.4%)	38 (41.8%)	
LYM (10^9^/L)	1.06 (0.81–1.45)	1.08 (0.84–1.37)	0.974
Normal (1.1–3.2 10^9^/L)	9 (50.0%)	44 (48.4%)	0.898
Abnormal	9 (50.0%)	47 (51.6%)	
MON (10^9^/L)	0.73 (0.45–0.81)	0.605 (0.43–0.93)	0.801
Normal (0.1–0.6 10^9^/L)	4 (22.2%)	19 (20.9%)	1.000
Abnormal	14 (77.8%)	72 (79.1%)	
RBC (10^12^/L)	3.21 (2.58–3.73)	3.1 (2.79–3.51)	0.769
Normal (3.8–5.1 10^12^/L)	4 (22.2%)	12 (13.2%)	0.532
Abnormal	14 (77.8%)	79 (86.8%)	
HGB (g/L)	99.39 ± 18.56	97.36 ± 15.91	0.631
Normal (115–150 g/L)	3 (16.7%)	11 (12.1%)	**0.596**
Abnormal	15 (83.3%)	80 (87.9%)	
PLT (10^9^/L)	156.9 (131.2–209.45)	168.75 (131.7–205.58)	0.695
Normal (125–350 10^9^/L)	10 (55.6%)	60 (65.9%)	**0.401**
Abnormal	8 (44.4%)	31 (34.1%)	
TP (g/L)	57.71 (49.44–63.93)	57.8 (54.18–61.6)	0.524
Normal (60–80 g/L)	6 (33.3%)	32 (35.2%)	0.882
Abnormal	12 (66.7%)	59 (64.8%)	
ALB (g/L)	34.49 (30.08–35.75)	34.23 (32.04–36.4)	0.562
Normal (35–55 g/L)	8 (44.4%)	37 (40.7%)	0.766
Abnormal	10 (55.6%)	54 (59.3%)	
GLOB (g/L)	23.30 (18.21–28.40)	23.4 (21.0–26.10)	0.488
Normal (20–30 g/L)	10 (55.6%)	72 (79.1%)	0.069
Abnormal	8 (44.4%)	19 (20.9%)	
A/G	1.52 ± 0.38	1.48 ± 0.32	0.623
Normal (1.0–2.5)	17 (94.4%)	86 (94.5%)	1.000
Abnormal	1 (5.6%)	5 (5.5%)	
CK (U/L)	179.2 (74.9–356.0)	112.5 (58.75–198.15)	0.209
Normal (25–130 U/L)	7 (38.9%)	49 (53.8%)	0.246
Abnormal	11 (61.1%)	42 (46.2%)	
CKMB (U/L)	10.51 (7.07–12.76)	10.95 (7.7–15.21)	0.618
Normal (3–20 U/L)	17 (94.4%)	35 (38.5%)	**< 0.0001**
Abnormal	1 (5.6%)	56 (61.5%)	
CRP (mg/L)	36.9 (13.0–69.5)	42.44 (17.49–75.74)	0.704
Normal (< 10 mg/L)	2 (11.1%)	11 (12.1%)	1.000
Abnormal	16 (88.9%)	80 (87.9%)	

Bold and italics just remind us the significant variables

Values are presented as the number (%) or the median (interquartile range). BMI are presented as mean or standard deviation

*DVT* deep venous thrombosis, *DG* distal group, *PG* proximal group, *PT* prothrombin time, *INR* international normalized ratio, *FIB* fibrinogen, *APTT* activated partial thromboplastin time, *TT* thrombin time, *AT III* antithrombin III, *WBC* white blood cell, *NEU* neutrophil, *LYM* lymphocyte, *MON* monocyte, *RBC* red blood cell, *HGB* hemoglobin, *PLT* platelet, *TP* total protein, *ALB* albumin, *GLOB* globulin, *CK* creatine kinase, *CKMB* creatine Kinase Isoenzyme, *CRP* c-reactive protein

*p < 0.05, statistical significance

## Discussion

IF, one of the most common fractures in elderly patients, can cause high risks of mortality and morbidity, as evidenced by previous studies [8, 15] as well as lead to a hypercoagulable state and immobilization that further result in DVT. It is well known that aDVT is an important contributor to delaying the time from admission to surgery. Although ongoing research has focused on the investigation of DVT after hip fractures [8, 15–19], including IF, femur neck fracture, and subtrochanteric fracture, less attention is paid on aDVT and a special population of nonagenarians and centenarians is rapidly growing along with the advancement of medical care.

To our knowledge, this is the first study concerning nonagenarians and centenarians to investigate the risk factors for aDVT and proximal aDVT after IF. In our study, the rate of aDVT was 34.3%, and 5.7% of patients had proximal aDVT. Logistic regression analysis showed that female patients and a high level of D-dimer were risk factors for aDVT. Similarly, hypoproteinemia and a high level of D-dimer were found to be risk factors for proximal aDVT. ROC curve analysis indicated the cut-off values of D-dimer to predict the aDVT and proximal aDVT were 1.28 mg/L and 1.485 mg/L, respectively.

Our findings showed the rates of aDVT (109 of 318) were 34.3%, including 5.7% (18 of 318) of patients with proximal aDVT and 28.6% (91 of 318) of patients with proximal aDVT. Zuo [16] reported 20.1% of patients who were older than 60 years old with aDVT after IF, while Zhao [11] retrospectively reviewed 1360 geriatric IF patients and found 10.2% of patients having preoperative DVT. Unquestionably, the older subjects included in the present study are closely related to a higher incidence of aDVT. From another perspective, this difference greatly proves that advanced age is an independent risk factor for aDVT and orthopedic surgeon should pay more attention on aDVT in nonagenarians and centenarians with IF, especially proximal aDVT that may be more likely to have PE.

D-dimer is widely used to help clinicians estimate the occurrence of DVT. However, it is susceptible to various variables, including inflammation, age, surgery, hospitalization, and other acute disorders [19–22], resulting in high sensitivity but low specificity. Therefore, it is urgent to identify the cut-off value of D-dimer based on age-stratified to diagnose DVT in the geriatric patients, particularly in HF patients. Kearon [23] considered D-dimer < 500 mg/L as a high predictive value due to pretest probability. The age-adjusted D-dimer threshold increased by 10 mg/L per 10 years for patients who were older than 50 years old. After reviewing studies that only focus on DVT after IF, our findings were similar to previous studies [11, 16] that a higher level of D-dimer was found in the aDVT group compared with the non-aDVT group. However, the cut-off value of D-dimer for predicting DVT was controversial. Zhao [11] used the ROC analysis to investigate the cut-off value of D-dimer and found > 1.59 mg/L as the optimal threshold for the diagnosis of preoperative DVT in geriatric IF patients. Zou [16] reported 1.44 mg/L as the cut-off value to judge the aDVT. However, our cut-off value of D-dimer was 1.28 mg/L to diagnose aDVT, which was lower than prior literature [11, 16]. Two possible factors can account for the discrepancy in the cut-off value. First, it is well known that the level of D-dimer closely depends on the time from injury to detection, implying that the different checking time of D-dimer can explain this discrepancy. We found patients receiving D-dimer test before surgery in the study of Zhao [11], which was later than ours. Second, the specificity of cut-off value (47.8%) in the study of Zou [16] was lower than ours (60.3%), implying a relatively high predictive value in our study. In this study, the optimal cut-off value of D-dimer for the diagnosis of proximal aDVT was 1.485 mg/L. However, its specificity remains low (49.5%), which could be used as an auxiliary indicator to improve diagnostic accuracy rate. Our findings firstly provide an optimal cut-off value of D-dimer, particularly for nonagenarians and centenarians following IF to the diagnosis of aDVT and proximal DVT, helping clinicians take timely preventive measures to reduce the time from admission to surgery.

Regarding hip fracture, Kobayashi [12] and Wang [8] performed a meta-analysis to find the predictive role of female patients in preoperative DVT, which was consistent with the result of Xing [17]. Xing [17] found that female patients had 68–73% sensitivity and 36–43% specificity in the diagnosis of preoperative DVT in Asian patients. However, in terms of IF, Zhao [11] and Zou [16] did not find close relationship between female patients and aDVT or preoperative DVT. These were inconsistent with our results, which may be related to the difference in the ages of the subjects. We found female patients were an independent risk factor for aDVT that may be partially associated with the genetic differences and hormonal changes after menopause and its associated complications [24, 25], but female patients were not a predictor for proximal aDVT that may be explained by the fact that female patients make up 85.3% of all aDVT patients. Previous studies [8, 11, 16] have demonstrated that patients with a history of hypoproteinemia were related to a risk of aDVT in hip fracture patients, which is consistent with our results. It might be explained by the hyperfibrinogenemia and platelet aggregability triggered by hypoalbuminemia [26, 27]. Similarly, in the present study, patients with a history of anemia were more likely to have preoperative DVT [28].

Although this study provides several novel findings, we should point out some limitations. This was a single-center study with limited samples, so we cannot perform subgroup analysis, such as gender. Therefore, a large sample, multicenter, and randomized controlled study is urgently needed. Secondly, some potential variables associated with the risks of aDVT cannot be fully identified due to this retrospective study, such as a history of smoking. Third, as with every other multivariate analysis, we could not include all confounding factors and residual confounding remains an issue.

In summary, we found that female patients, patients with a history of anemia, and the level of D-dimer were risk factors for aDVT, as well as hypoproteinemia and the level of D-dime were independent predictors of proximal aDVT. We also identified 1.28 mg/L and 1.485 mg/L as the cut-off values of D-dimer to predict the aDVT and proximal aDVT, respectively. It is important to reduce perioperative complications, such as DVT, under the multidisciplinary project to lower morbidity and mortality. Our findings provide individualized assessment risk of aDVT and proximal aDVT for nonagenarians and centenarians with intertrochanteric fractures to manage early targeted interventions.

## Data Availability

Yes.

## Abbreviations

aDVT: Admission deep venous thrombosis
IF: Intertrochanteric fracture
DVT: Deep venous thrombosis
PE: Pulmonary embolism
DG: Distal group
PG: Proximal group
PT: Prothrombin time
INR: International normalized ratio
FIB: Fibrinogen
APTT: Activated partial thromboplastin time
TT: Thrombin time
AT III: Antithrombin III
WBC: White blood cell
NEU: Neutrophil
LYM: Lymphocyte
MON: Monocyte
RBC: Red blood cell
HGB: Hemoglobin
PLT: Platelet
TP: Total protein
ALB: Albumin
GLOB: Globulin
CK: Creatine kinase
CKMB: Creatine Kinase Isoenzyme
CRP: C-reactive protein
